# Disorders of sex development: insights from targeted gene sequencing of a large international patient cohort

**DOI:** 10.1186/s13059-016-1105-y

**Published:** 2016-11-29

**Authors:** Stefanie Eggers, Simon Sadedin, Jocelyn A. van den Bergen, Gorjana Robevska, Thomas Ohnesorg, Jacqueline Hewitt, Luke Lambeth, Aurore Bouty, Ingrid M. Knarston, Tiong Yang Tan, Fergus Cameron, George Werther, John Hutson, Michele O’Connell, Sonia R. Grover, Yves Heloury, Margaret Zacharin, Philip Bergman, Chris Kimber, Justin Brown, Nathalie Webb, Matthew F. Hunter, Shubha Srinivasan, Angela Titmuss, Charles F. Verge, David Mowat, Grahame Smith, Janine Smith, Lisa Ewans, Carolyn Shalhoub, Patricia Crock, Chris Cowell, Gary M. Leong, Makato Ono, Antony R. Lafferty, Tony Huynh, Uma Visser, Catherine S. Choong, Fiona McKenzie, Nicholas Pachter, Elizabeth M. Thompson, Jennifer Couper, Anne Baxendale, Jozef Gecz, Benjamin J. Wheeler, Craig Jefferies, Karen MacKenzie, Paul Hofman, Philippa Carter, Richard I. King, Csilla Krausz, Conny M. A. van Ravenswaaij-Arts, Leendert Looijenga, Sten Drop, Stefan Riedl, Martine Cools, Angelika Dawson, Achmad Zulfa Juniarto, Vaman Khadilkar, Anuradha Khadilkar, Vijayalakshmi Bhatia, Vũ Chí Dũng, Irum Atta, Jamal Raza, Nguyen thi Diem Chi, Tran Kiem Hao, Vincent Harley, Peter Koopman, Garry Warne, Sultana Faradz, Alicia Oshlack, Katie L. Ayers, Andrew H. Sinclair

**Affiliations:** 1 Murdoch Childrens Research Institute, Melbourne, VIC Australia; 2 Department of Paediatrics, University of Melbourne, Melbourne, VIC Australia; 3 The Royal Children’s Hospital Melbourne, Melbourne, VIC Australia; 4 Victorian Clinical Genetic Services, Melbourne, VIC Australia; 5 University of Melbourne, School of Bioscience, Melbourne, VIC Australia; 6 Department of Paediatric Endocrinology and Diabetes, Monash Children’s Hospital, Clayton, VIC Australia; 7 Monash Medical Centre, Clayton, VIC Australia; 8 Monash Children’s Hospital, Clayton, VIC Australia; 9 Department Of Paediatric Urology, Monash Children’s Hospital, Clayton, VIC Australia; 10 Department of Paediatrics, Monash University, Clayton, VIC Australia; 11 Monash Genetics, Monash Health, Clayton, VIC Australia; 12 The Children’s Hospital at Westmead, Institute of Endocrinology and Diabetes, Westmead, NSW Australia; 13 Sydney Children’s Hospital, Randwick, NSW Australia; 14 School of Women’s and Children’s Health, UNSW, Sydney, NSW Australia; 15 Department of Medical Genetics, Sydney Children’s Hospital, Randwick, NSW Australia; 16 Urology and Clinical Programs, The Children’s Hospital at Westmead, Westmead, NSW Australia; 17 The University of Sydney, Westmead, NSW Australia; 18 Department of Clinical Genetics, The Children’s Hospital at Westmead, Westmead, NSW Australia; 19 Department of Medical Genomics, Royal Prince Alfred Hospital, Camperdown, NSW Australia; 20 Genetic Medicine, Sydney Medical School, University of Sydney, Sydney, NSW Australia; 21 Hudson Institute of Medical Research, Clayton, VIC Australia; 22 John Hunter Children’s Hospital, New Lambton Heights, NSW Australia; 23 Department of Paediatrics, Tokyo Bay Medical Centre, Tokyo, Chiba Japan; 24 Institute for Molecular Bioscience, The University of Queensland, Brisbane, QLD Australia; 25 Department of Paediatric Endocrinology and Diabetes, Lady Cilento Children’s Hospital, Brisbane, QLD Australia; 26 Centenary Hospital for Women and Children, Canberra, ACT Australia; 27 ANU Medical School, Canberra, ACT Australia; 28 Department of Endocrinology and Diabetes, Princess Margaret Hospital, Subiaco, WA Australia; 29 School of Paediatrics and Child Health, The University of Western Australia, Crawley, WA Australia; 30 Genetic Services of Western Australia, King Edward Memorial Hospital, Subiaco, WA Australia; 31 SA Clinical Genetics Service, SA Pathology at the Women’s and Children’s Hospital, North Adelaide, SA Australia; 32 School of Medicine, University of Adelaide, North Terrace, Adelaide, SA Australia; 33 Women’s and Children’s Hospital and Robinson Research Institute, University of Adelaide, Adelaide, SA Australia; 34 School of Medicine and The Robinson Research Institute, The University of Adelaide, Adelaide, SA Australia; 35 South Australian Health and Medical Research Institute, Adelaide, SA Australia; 36 Department of Women’s and Children’s Health, University of Otago, Dunedin, New Zealand; 37 Diabetes and Endocrinology, Auckland District Health Board, Auckland, New Zealand; 38 Christchurch Hospital, Christchurch, New Zealand; 39 Liggins Institute, University of Auckland, Auckland, New Zealand; 40 Starship Paediatric Diabetes and Endocrinology, Auckland, New Zealand; 41 Canterbury Health Laboratories, Christchurch, Canterbury New Zealand; 42 Department of Experimental and Clinical Biomedical Sciences “Mario Serio”, University of Florence, Florence, Italy; 43 University of Groningen, University Medical Centre Groningen, Groningen, Netherlands; 44 Department of Pathology, Josephine Nefkens Institute, Erasmus University Medical Centre, Rotterdam, Netherlands; 45 Department of Paediatrics, Division of Endocrinology, Sophia Children’s Hospital, Erasmus University Medical Centre, Rotterdam, Netherlands; 46 St Anna Children’s Hospital, Vienna, Austria; 47 Paediatric Department, Medical University of Vienna, Vienna, Austria; 48 Department of Paediatric Endocrinology, Ghent University Hospital, Ghent, Belgium; 49 Genomic Laboratory, Diagnostic Services of Manitoba and Genetics & Metabolism Program, WRHA, Winnipeg, MB Canada; 50 Department Biochemistry & Medical Genetics and Paediatrics & Child Health, University of Manitoba, Winnipeg, MB Canada; 51 Division of Human Genetics, Centre for Biomedical Research Faculty of Medicine Diponegoro University (FMDU), Semarang, Indonesia; 52 Growth and Pediatric Endocrine Clinic, Hirabai Cowasji Jehangir Medical Research Institute, Pune, India; 53 Hirabai Cowasji Jehangir Medical Research Institute, Pune, India; 54 Department of Endocrinology Sanjay Gandhi PGI, Lucknow, India; 55 Department of Endocrinology, Metabolism and Genetics National Children’s Hospital, Hanoi, Vietnam; 56 National Institute of Child Health, Karachi, Pakistan; 57 Paediatric Centre, Hue Central Hospital, Hue city, Vietnam

**Keywords:** Disorders of sex development, Gonad, Testis, Ovaries, Ovotestes, Massively parallel sequencing, MPS, Cohort, Targeted gene panel, Genetic diagnosis, Variant, Mutation

## Abstract

**Background:**

Disorders of sex development (DSD) are congenital conditions in which chromosomal, gonadal, or phenotypic sex is atypical. Clinical management of DSD is often difficult and currently only 13% of patients receive an accurate clinical genetic diagnosis. To address this we have developed a massively parallel sequencing targeted DSD gene panel which allows us to sequence all 64 known diagnostic DSD genes and candidate genes simultaneously.

**Results:**

We analyzed DNA from the largest reported international cohort of patients with DSD (278 patients with 46,XY DSD and 48 with 46,XX DSD). Our targeted gene panel compares favorably with other sequencing platforms. We found a total of 28 diagnostic genes that are implicated in DSD, highlighting the genetic spectrum of this disorder. Sequencing revealed 93 previously unreported DSD gene variants. Overall, we identified a likely genetic diagnosis in 43% of patients with 46,XY DSD. In patients with 46,XY disorders of androgen synthesis and action the genetic diagnosis rate reached 60%. Surprisingly, little difference in diagnostic rate was observed between singletons and trios. In many cases our findings are informative as to the likely cause of the DSD, which will facilitate clinical management.

**Conclusions:**

Our massively parallel sequencing targeted DSD gene panel represents an economical means of improving the genetic diagnostic capability for patients affected by DSD. Implementation of this panel in a large cohort of patients has expanded our understanding of the underlying genetic etiology of DSD. The inclusion of research candidate genes also provides an invaluable resource for future identification of novel genes.

**Electronic supplementary material:**

The online version of this article (doi:10.1186/s13059-016-1105-y) contains supplementary material, which is available to authorized users.

## Background

Disorders of sex development (DSD) are defined as congenital conditions in which the chromosomal, gonadal, or phenotypic sex is atypical [1]. This group of disorders are highly heterogeneous and include clinical phenotypes such as hypospadias (misplacement of the urethral meatus; 1 in 250 boys), ambiguous genitalia (1 in 4500 live births), and complete XX or XY sex reversal (1 in 20,000 births) [2–4] (reviewed in [5]). DSD represent a major pediatric concern and a significant healthcare burden due to the difficult clinical management of these conditions and, in some, the association with gonadal cancer and infertility. Uncertainty about a child’s gender can be extremely traumatic for the individual, parents, and other family members and may carry profound psychological and reproductive consequences for the patient. Most often the underlying cause of DSD is a variant in a gene or genes regulating gonadal/genital or steroidogenic pathways.

Providing a molecular diagnosis for patients with a DSD and families can serve multiple purposes: naming the underlying cause contributes to acceptance, reduces stigma or blame, and provides crucial clues and guidance for clinical management, including information on the malignancy risks associated with some types of DSD [6]. A diagnosis is integral to genetic counseling and family planning and yet it has been found that as few as 13% of patients with a DSD will receive a clinical molecular genetic diagnosis in the current hospital system [7].

Massively parallel sequencing (MPS) has been widely adopted for the diagnosis of genetic diseases, especially for monogenic congenital disorders, as it promises to improve diagnosis and alter patient management through rapid sequencing of many genes simultaneously at a lower cost compared with sequential testing of multiple genes. The process of deploying these genomic assays involves extensive evaluation of technology, bioinformatics, and clinical concerns to choose the right configuration for a given setting. As technology advances and whole genome sequencing (WGS) or whole exome sequencing (WES) becomes more accessible, the choice of platform must take both performance and cost into consideration. In some countries either government or private health insurance funding covers or contributes to the cost of WES to diagnose DSD patients, and this has been reported for a number of individuals affected by 46,XY DSD [8]. In Australia, however, MPS is not yet covered by the national Medicare system or private health insurance bodies. In this environment, an MPS targeted gene panel offers many advantages, such as relatively low cost, shorter turnaround time, and smaller overheads in data handling and analysis compared to WES or WGS. Indeed, numerous gene panels have been successfully employed in the genetic diagnosis of a variety of monogenic disorders [9], including small cohorts of patients with 46,XY DSD [7, 10]. Finally, no studies have reported the usefulness of MPS for patients with 46,XX DSD, nor have any large-scale studies looked at the contribution of known DSD genes to this heterogeneous condition.

Here, we report the application of an MPS targeted gene panel to a cohort of patients affected by DSD (both 46,XX and 46,XY DSD). This panel contains genes of both clinical and research relevance that are associated with gonadal or genital development as well as steroidogenic pathways. It includes the majority of known diagnostic genes for DSD, allowing us to perform the same diagnostic test on all DSD patients and their participating family members irrespective of their DSD phenotype. Performance evaluation of our MPS targeted DSD gene panel in comparison to both WGS and well-characterized reference samples shows that it offers high sensitivity and specificity. The results from targeted genetic testing of 326 patients with DSD (and 129 of their family members) from a wide spectrum of clinical presentations (the largest known such cohort) are presented.

## Results

### A targeted DSD gene panel: performance evaluation

We designed a targeted gene panel for DSD using HaloPlex (Agilent) technology. This system allowed us to simultaneously sequence 64 known diagnostic genes for DSD and an additional 967 candidate genes. HaloPlex technology uses custom molecular inversion probes (SureDesign software, Agilent) that are then used for selective circularization-based target enrichment. The known diagnostic genes have been compiled from current knowledge of DSD sourced from PubMed and clinical variant databases (such as HGMD and ClinVar) (Table 1). The candidate genes included in the panel were selected from several sources, including research studies reporting candidate DSD genes, genes implicated in gonadal development from animal models, RNA-seq studies, and known molecular pathways (such as Hedgehog signaling, WNT signaling, and androgen receptor (AR) interacting proteins). In addition we have included relevant regulatory regions and microRNAs, which are not possible to detect using WES. This manuscript reports only variants found in the 64 diagnostic DSD genes; however, ongoing work in our research group is addressing the contribution of candidate genes to DSD.

**Table 1:** Diagnostic DSD genes included in the panel

	Gene	Locus	OMIM	Associated DSD	Inheritance	Coverage (>20×)
Gonadal development						
	*BMP15*	Xp11.22	300247	46,XX DSD—ovarian dysgenesis	AD	95%
	*CBX2*	17q25.3	602770	46,XY DSD CGD	AR	96%
	*DHH*	12q13.12	605423	46XY PGD or CGD	AR, AD	99%
	*DMRT1*	9p24.3	602424	46,XY DSD	AD: deletion	98%
	*DMRT2*	9p24.3	604935	46,XY DSD	AD: deletion	99%
	*FOXL2*	3q22.3	608996	POI alone or with blepharophimosis, ptosis, and epicanthus inversus syndrome	AD	94%
	*GATA4*	8p23.1	600576	46,XY DSD	AD	91%
	*NR0B1*	Xp21.2	300473	46,XY GD—gain of function	XL-dup	100%
				46,XX CAH with HH	XLR	
	*NR5A1*	9q33.3	184757	46,XY DSD (various)	AD	100%
				46,XX POI	AD	
	*MAP3K1*	5q11.2	600982	46,XY GD	AD	99%
	*RSPO1*	1p34.3	609595	46,XX OT DSD with palmoplantar hyperkeratosis	AR	94%
	*SOX3*	Xq27.1	313430	46,XX T or OT DSD—gain of function	XL: dup	92%
	*SOX9*	17q24.3	608106	46,XY GD and campomelic dysplasia	AD	95%
				46,XX T DSD—duplication	AD: dup	
	*SRY*	Yp11.2	480000	46,XX T DSD—gain of function	Translocation	58%*
				46,XY ovarian DSD	AD	
	*TSPYL1*	6q22.1	604714	46,XY DSD with sudden infant death syndrome	AR	99%
	*WNT4*	1p36.12	603490	46,XY ovo or OT DSD or 46,XY CGD—duplication	AD: dup	99%
				46,XX T DSD	AR	
				46,XX MRKH	AD	
	*WT1*	11p13	607102	Frasier syndrome and Denys-Drash	AD	99%
	*ZFPM2*	8q23.1	603693	46,XY GD	AD	99%
Gonadal differentiation (androgen synthesis and action)						
	*AKR1C2*	10p15.1	600450	46,XY DSD	AR	82%
	*AKR1C4*	10p15.1	600451	46,XY DSD	AR	99%
	*AMH*	19p13.3	600957	PMDS	AR	94%
	*AMHR2*	12q13.13	600956	PMDS	AR	100%
	*AR*	Xq12	313700	46,XY DSD. Complete AIS/partial AIS, isolated hypospadia	XL	97%
	*ARX*	Xp21.3	300215	X-linked lissencephaly with ambiguous genitalia	XL	90%
	*ATRX*	Xq21.1	300032	46,XY DSD associated with alpha-thalassemia X-linked intellectual disability syndrome	XL	99%
	*CDKN1C*	11p15.4	600856	Genital anomalies in association with Beckwith-Wiedemann and IMAGE syndrome	AD	61%
	*CYB5A*	18q22.3	613218	46,XY DSD	AR	99%
	*CYP11A1*	15q24.1	118485	46,XY sex reversal (partial or complete) with adrenal insufficiency. CAH	AR	100%
				Hypospadias	AD	
	*CYP11B1*	8q24.3	610613	46,XX DSD. CAH due to steroid 11-beta-hydroxylase deficiency	AR	86%
	*CYP17A1*	10q24.32	609300	46, XY DSD. 17,20-lyase deficiency CAH	AR	100%
	*CYP19A1*	15q21.2	107910	46, XY DSD. Aromatase deficiency	AR	100%
	*CYP21A2*	6p21.33	613815	46, XX DSD virilization—21-hydroxylase-deficient CAH	AR	6%
	*FGFR2*	10q26.13	176943	46,XY GD with craniosynotosis. Apert syndrome	AD	99%
	*HSD17B3*	9q22.32	605573	46,XY DSD—17-β-hydroxysteroid dehydrogenase III deficiency	AR	100%
	*HSD17B4*	5q23.1	233400	Perrault syndrome (with ovarian dygeneisis in 46,XX)	AR	98%
	*HSD3B2*	1p12	613890	46,XY DSD and 46,XX DSD—3-β-hydroxysteroid dehydrogenase-deficient CAH	AR	99%
				Hypospadias	AD	
	*LHCGR*	2p16.3	152790	46,XY DSD—Leydig cell hypoplasia,	AR	100%
				Precocious puberty (male)	AD	
	*NR3C1*	5q31.3	138040	46,XX hyperandrogenism	AD	96%
	*POR*	7q11.23	124015	Cytochrome P450 oxidoreductase deficiency	AR	95%
	*SRD5A2*	2p23.1	607306	46,XY DSD. Steroid 5-α-reductase deficiency	AR	100%
				Hypospadias	AD	
	*STAR*	8p11.23	600617	46,XY DSD—cholesterol desmolase-defient CAH	AR	100%
Central causes of hypogonadism						
	*BBS9*	7p14.3	615986	Bardet-Biedl syndrome	AR	96%
	*CHD7*	8q12.2	608892	CHH or KS. CHARGE syndrome	AD	99%
	*FGF8*	10q24.32	612702	CHH or KS	AD	88%
	*FGFR1*	8p11.23	147950	CHH or KS	AD	100%
	*FSHB*	11p14.1	136530	CHH	AD	98%
	*FSHR*	2p16.3	136435	46,XX ovarian dysgenesis	AR	100%
	*GNRH1*	8p21.2	152760	CHH	AR	100%
	*GNRHR*	4q13.2	138850	CHH	AR	100%
	*HESX1*	3p14.3	601802	KS or CPHD	AD	98%
	*KAL1*	Xp22.31	300836	CHH or KS	XL	97%
	*KISS1R*	19p13.3	604161	CHH or KS	AD	96%
	*LEP*	7q32.1	164160	CHH with obesity	AR	99%
	*LHX3*	9q34.3	600577	CPHD	AR	89%
	*PROK2*	3p13	607002	CHH or KS	AD	99%
	*PROKR2*	20p12.3	607123	CHH or KS	AD	100%
	*PROP1*	5q35.3	601538	CPHD	AR	98%
	*TAC3*	12q13.3	162330	CHH	AR	98%
	*WDR11*	10q26.12	606417	CHH or KS	AD	93%
Other (isolated hypospadia, cryptorchidism, MRKH):						
	*ATF3*	1q32.3	603148	46,XY isolated hypospadias	AD	97%
	*HOXA13*	7p15.2	142959	Hand-foot uterus syndrome - MRKH in 46,XX	AD	93%
				Guttmacher syndrome in 46,XY including hypospadias	AD	
	*INSL3*	19p13.11	146738	Cryptorchidism	AD	99%
	*MAMLD1*	Xq28	300120	Hypospadias	XL	100%
	*RXFP2*	13q13.1	606655	Cryptorchidism	AD	96%

DSD genes considered as diagnostic that were included in the targeted gene panel. These are grouped according to their main action during development (gonadal development, androgen or hormonal activity, central causes of hypogonadism or other). Gene locus is shown as well as reference number for OMIM (Online Mendelian Inheritance in Man). The associated DSD(s) are shown for each gene. *CGD* complete gonadal dysgenesis, *PGD* partial gonadal dysgenesis, *POI* premature ovarian insufficiency, *GD* gonadal dysgenesis, *CAH* congenital adrenal hyperplasia, *HH* hypogonadotrophic hypogonadism, *OT* ovo-testicular, *T* testicular, *MRKH* Mayer-Rokitansky-Küster-Hauser syndrome, *PMDS* persistent Müllerian duct syndrome, *AIS* androgen insensitivity syndrome, *CHH* congenital hypogonadotrophic hypogonadism, *KS* Kallmann syndrome, *CPHD* central pituitary hormone defect. Mode of inheritance is shown: *AR* autosomal recessive, *AD* autosomal dominant, *XL* X-linked, *dup* duplication (gain of function). The percentage coverage with greater than 20× depth is also shown

To provide a benchmark of assay quality, we created an evaluation data set that included 16 samples, three of which had been previously sequenced using WGS. These 16 samples were sequenced using our targeted gene panel on a single run using an Illumina MiSeq instrument, configured to produce 2 × 150-bp paired-end reads.

This dataset was evaluated to ascertain performance of the panel with respect to several standard benchmarks for MPS assays, including coverage, targeting efficiency, and variant calling accuracy.

#### Coverage

**Fig. 1:**
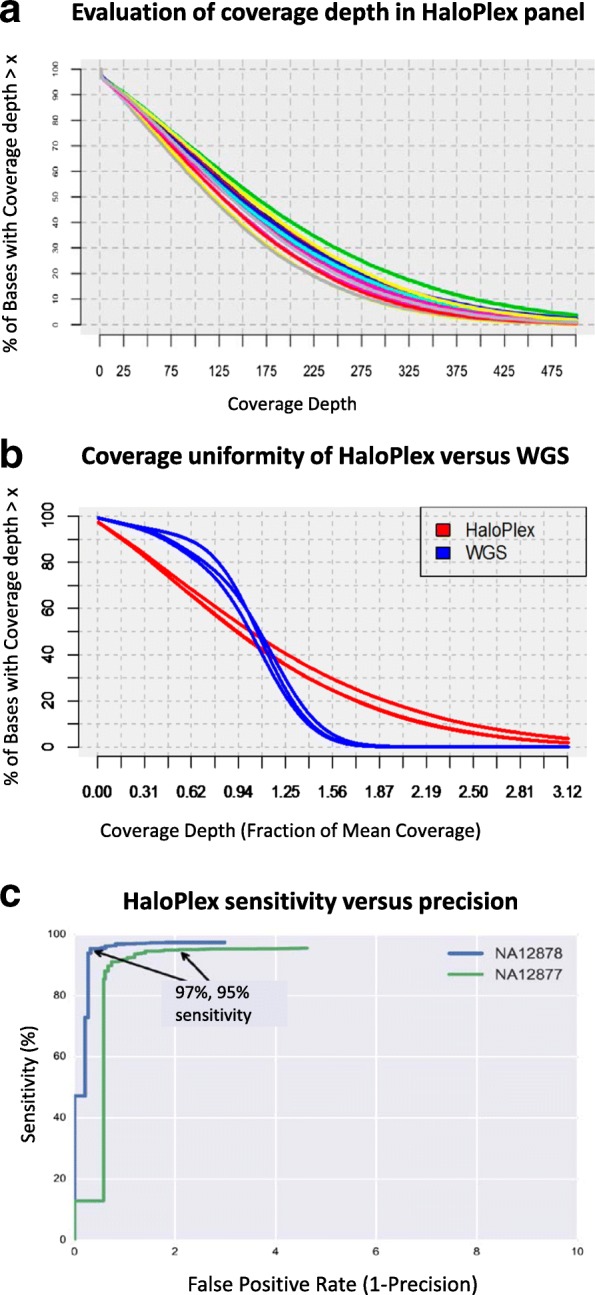
Coverage and variant properties of the panel and patient cohort. **a** The cumulative distribution read coverage across the targeted regions of the HaloPlex panel for 16 evaluation samples. The *vertical axis* shows the percentage of bases covered with at least the level of coverage specified by the *horizontal axis*. Although the median coverage is acceptable for all samples, it is notable that 10% of bases are covered at less than 25×, while another 10% of bases are covered at more than 280×. **b** Coverage depth uniformity of HaloPlex compared to whole genome sequencing (*WGS*). The cumulative coverage distribution is shown for three samples sequenced by both technologies. HaloPlex is notably less uniform, having a flatter distribution than WGS. **c** Receiver-operator characteristic (ROC) curve showing sensitivity versus false positive rate (1 − precision) for detecting single nucleotide variants and INDELs smaller than 10 bp, compared to high confidence calls for samples NA12878 and NA12877. Call sets were obtained from the Illumina Platinum Genomes project. A sensitivity of 97 and 95%, respectively, is achieved for a false positive rate smaller than approximately 2% in both cases

A commonly accepted threshold for variant calling is approximately 30× in research settings, while higher thresholds are frequently sought for diagnostic use. In aggregate, the targeted gene sequencing of our evaluation data set yielded mean (median) coverage depths well in excess of these thresholds, varying between 135× (115×) and 190× (161×). However, the coverage depth was highly variable across different genomic regions. Approximately 10% of bases were covered at lower than 30× and the upper 10% of bases were covered at greater than 280× (Fig. 1a). WGS showed more even coverage, with 90% of bases having at least half of the mean coverage compared with only 70% of bases having half the mean coverage for our targeted panel (Fig. 1b). Nonetheless, coverage uniformity of our targeted gene panel (HaloPlex) is approximately similar to that cited when comparing other targeted capture technologies, including WES [11].

#### Targeting efficiency

Averaged across the evaluation samples, we observed that 92% of sequenced fragments overlapped the target region by at least 1 bp. This percentage compares favorably with commonly cited targeting accuracy for competing platforms such as Agilent SureSelect and Nimblegen [12]. However, we also observe that a substantial proportion of the reads overlap the targeted regions by only a small amount. If targeting efficiency is calculated at the base level, only 66% of sequenced bases overlapped the targeted regions, significantly reducing the overall efficiency.

#### Adapter contamination

We found that a high fraction of reads experienced “read through” into adapters, resulting in numerous high confidence false positive variant detections when analysis was run using raw data. A satisfactory compromise between over-trimming (trimming of non-adapter sequence) and under-trimming (substantial adapter contamination remaining in the data) was not achieved using a number of tools, including Trimmomatic [13], SeqPrep (https://github.com/jstjohn/SeqPrep) and Agilent’s MPS ReadTrimmer (http://download.chem.agilent.com/software/ngs_readtrimmer). Thus, a custom trimming program was designed, resulting in nearly 100% of reads being correctly trimmed of adapter sequences (see “Methods”).

#### Underperforming amplicons

Performance of our targeted gene panel at any given genomic locus is critically dependent on the performance of the handful of amplicons that span the locus. The 29,928 amplicons in our evaluation design showed highly variable performance, including a substantial number of amplicons (8% on average) to which no reads are mapped. Some of these “failures” occurred consistently between samples: 38% of amplicons that failed did so in all of our evaluation samples. However, we also observed that 13% of failures occurred sporadically, in only a single sample.

#### Variant calling accuracy

We evaluated variant calling accuracy using two independent data sets: firstly, the three samples sequenced independently using WGS offer a comparison to a technology free of bias due to the targeted capture process. Secondly, we sequenced a trio (NA12877, NA12878, NA12879) of samples from the 1000 Genomes CEPH pedigree. These samples have been intensively studied and sets of gold standard variant calls are available for comparison from the Illumina Platinum Genomes Project (http://www.illumina.com/platinumgenomes/). In comparison to the gold standard reference call set, we observed high sensitivity and specificity of our targeted gene panel. At a false positive rate of 2%, variant calls for NA12878 and NA12877 achieved an overall sensitivity of 97% (for 974 variant calls) and 95% (for 1278 variants calls), respectively. Variant calls were compared using the RTG vcfeval (http://realtimegenomics.com/products/rtg-tools/Cleary2015) utility for single nucleotide changes and INDELs smaller than 10 bp (Fig. 1c). In the case of our samples that were also sequenced using WGS, we manually scrutinized differences between variant calls obtained from our targeted gene panel and WGS data to ascertain the likely cause for each discrepancy. The predominant reason for false negatives in our panel variant calls was due to the amplicon design. That is, in 63% of cases either no amplicon was present over the region or the amplicons produced insufficient coverage depth to call a variant. False positives in our targeted gene panel data occurred due to either systematic misalignment of a particular amplicon or to regions of poor sequencing quality that generated large numbers of sequencing errors. In both cases the errors were systematically confined to narrow genomic loci and thus could be eliminated bioinformatically.

### A large international cohort of patients with DSDs

We have assembled DNA from the largest known international cohort of patients affected by DSD. A total of 326 patients with a DSD were included in this sequencing analysis (Table 2). This included 251 patients sequenced as singletons and 75 patients with family members (129 family members, duos/trios or siblings; Table 2). We have classified the cohort of patients according to the 2006 Consensus Statement on Management of Intersex Disorders [1] (Table 2). Given the large number of patients, detailed clinical notes are beyond the scope of this meta-analysis and have only been provided where a patient is discussed in detail. It is important to note that persons with a known genetic etiology for sex chromosome disorders, as well as those with congenital adrenal hyperplasia (CAH), were not included in this study.

**Table 2:** Disorder of sex development patient cohort and variant summary

Cohort					DSD gene variants		Variant classification (number of patients)		
Classification		Trios/duos	Singletons	Total	Patients with no DSD variant	Patients with curated variant	Pathogenic	Likely pathogenic	VUS
**46,XY DSD**									
(A) Disorders of gonadal (testicular) development									
46,XY	Complete gonadal dysgenesis (CGD)	3	21	24	11	13	6	7	0
46,XY	Partial gonadal dysgenesis (PGD)	2	19	21	13	8	2	4	2
46,XY	Ovo-testicular DSD (OT)	3	3	6	4	2	1	1	0
46,XY	Gonadal regression (GR)	0	1	1	1	0	0	0	0
				**52**	**29**	**23**	Genetic diagnosis = 21 (40%)		
(B) Disorders in androgen synthesis or action									
46,XY	Suspected androgen syn/action disorder (DASA)	12	23	35	11	24	18	2	4
46,XY	Leydig cell hypoplasia (LCH)	0	1	1	0	1	0	1	0
46,XY	Persistent mullerian duct syndrome (PMDS)	1	0	1	0	1	1	0	0
				**37**	**11**	**26**	Genetic diagnosis = 22 (60%)		
(C) Other									
46,XY	Hypospadias	12	34	46	20	26	6	10	10
46,XY	Syndromic	5	4	9	6	3	1	1	1
46,XY	Diphallus	0	1	1	1	0	0	0	0
				**56**	**27**	**29**	Genetic diagnosis = 18 (32%)		
(D) Unknown									
46,XY	DSD (origin unknown)	25	108	133	52	81	41	16	24
							Genetic diagnosis = 57 (43%)		
**Total 46,XY DSD**		**63**	**215**	**278**	**119**	**159**	**76**	**42**	**41**
							**Genetic diagnosis = 118 (43%)**		
**46,XX DSD**									
(A) Disorders of gonadal (ovarian) development									
46,XX	Testicular DSD	2	14	16	8	8	8	0	0
46,XX	Ovotesticular DSD	2	5	7	6	1	0	0	1
46,XX	Gonadal dysgenesis (GD)	2	1	3	3	0	0	0	0
				**26**	**17**	**9**	Genetic diagnosis = 8 (31%)		
(B) Androgen excess				0					
(C) Other									
46,XX	MRKH	0	9	9	9	0	0	0	0
46,XX	Dysplastic ovaries	1	0	1	1	0	0	0	0
46,XX	Syndromic	1	1	2	2	0	0	0	0
				**12**	**12**	**0**			
(D) Unknown									
46,XX	DSD (origin unknown)	4	6	10	10	0	0	0	0
**Total 46,XX DSD**		**12**	**36**	**48**	**39**	**9**	**8**	**0**	**1**
							**Genetic diagnosis = 8 (17%)**		
**Total all DSD**		**75**	**251**	**326**	**158**	**168**	**84**	**42**	**42**

Patients have been categorized based on clinical notes provided, according to the recommended classification of DSD in the Chicago Consensus report [1]. The number of singleton and patients with family members (duos or trios) are shown for each DSD. Given the difficulty of classifying some DSD patients, we have included an “unknown” category for 46,XY undervirilized patients and 46,XX virilized patients with unknown cause. This table also lists the numbers of patients in each DSD classification with a variant in a clinically relevant DSD gene. This is shown as a total number and broken down into variant classifications: pathogenic, likely pathogenic, and variants of uncertain significance (VUS). Variants which are classified as pathogenic or likely pathogenic are considered to be a genetic diagnosis and have been indicated for each phenotypic category. In cases where a patient had variants in multiple genes, the variant with the highest classification (pathogenic > likely pathogenic > VUS) was taken into consideration for this chart. The exact variants in each patient can be found in Additional file 1: Table S1. Entries in bold are subtotals and totals. *MRKH* Mayer-Rokitansky-Küster-Hauser syndrome

Of the 326 patients, 278 were classified as having 46,XY DSD based on previous chromosomal karyotyping and clinical presentation (Table 2). These include 24 patients with 46,XY complete gonadal dysgenesis (CGD), 21 with 46,XY partial gonadal dysgenesis (PGD), and six with 46,XY ovotesticular DSD (OT). These patients have been classified as having a disorder of gonadal (testicular) development (Table 2). Furthermore, we have 37 46,XY DSD patients with a suspected disorder in androgen synthesis and action (DASA). Another 56 patients have been classified as having 46,XY DSD “other”, including 46 with hypospadias and one with diphallus/cloacal anomaly (Table 2). An additional 133 patients were defined as having a 46,XY DSD of unknown origin —broadly referring to those with varying degrees of under-virilization phenotypes, such as micropenis, cryptorchidism, and non-isolated hypospadias for which the underlying cause was unknown.

We also have DNA samples from 48 patients with 46,XX DSD (including 12 with family members). This cohort includes 26 patients with a disorder of gonadal (ovarian) development, including seven with 46,XX OT DSD, 16 with testicular (T) DSD, and three with gonadal dysgenesis. Nine individuals with 46,XX Mayer-Rokitansky-Küster-Hauser syndrome (MRKH) and one with dysplastic ovaries were also included. In addition, we have DNA from ten patients with 46,XX virilization of unknown origin (Table 2). Finally, 11 patients (46,XY and 46,XX) who have been referred with a DSD as part of a wider spectrum of anomalies, classified as syndromic DSD, were included (Table 2). To our knowledge around 30% of the cohort (both singletons and trios) had undergone pre-screening prior to participating in this study, such as single-gene Sanger sequencing (for example *AR*, *SRD5A2*, *HSD17B3*, *SRY*, *DHH*, or *WT1*).

Our cohort of patients with DSD covers 12 countries including Australia (83), New Zealand (7), Indonesia (97), the Netherlands (38), Pakistan (25), Vietnam (35), Cambodia (16), Austria (15), Belgium (6), Canada (2), India (1), and Italy (2).

### General characteristics of observed variants

Prior to filtering, 1,097,162 variants were observed in the entire cohort of patient samples in diagnostic genes and research candidates. Of these variants, 48% were observed recurrently in the cohort, with the total set comprising only 57,320 unique variants; 12,257 variants were novel (unseen in ESP6500, dbSNP, ExAC, or the 1000 Genomes Project) and 23% of novel variants were observed recurrently in our samples and were interpreted as either sequencing artifacts or common population variants that are endemic to specific ethnicities in our cohort. These are largely removed by our variant filtering process (see “Methods”). The majority (88%) of the protein changing variants observed in diagnostic genes were characterized as missense. Protein changing INDELs were dominated by inframe INDELs (14, 67%) followed by 1-bp or 2-bp frameshift variants (11, 28%). Only two frameshift INDELs larger than 2 bp were detected in the diagnostic gene set. The predominance of inframe INDELs is consistent with a high level of selection against significant disruption to these genes. However, the lack of observation of larger INDELs may be partly due to insensitivity of the analysis to longer INDELs.

### Diagnostic DSD gene coverage and calling

Coverage of clinically diagnostic genes for DSD is of critical interest and indicates potential utility of the panel as a diagnostic assay. In our evaluation data set, the design covered 99.4% of the bases within the targeted regions of these genes with at least one amplicon, while 97.2% of bases were covered with two or more amplicons. We evaluated total coverage of each DSD gene in 100 representative patient samples (from three separate library preparations). All genes except six had at least 90% coverage at 20× or greater (Table 1). Those lower than 90% were *SRY* (a Y-chromosome linked gene which is lower in this calculation because of the inclusion of both females and males), *AKR1C2*, *CDKN1C*, *CYP11B1*, *FGF8*, *LHX3*, and *CYP21A2* (82, 61, 86, 88, 89, and 6%, respectively) (Table 1). In some cases, large regions of these genes were covered at less than 20× coverage depth. For *CYP21A2*, low mappability of reads is caused by the presence of a pseudogene with very high sequence homology. Pathogenic variants in *CYP21A2* are thought to underlie up to 90–95% of CAH [14]. However, given our inability to confidently call variants in this gene, we have excluded CAH patients from our cohort.

**Fig. 2:**
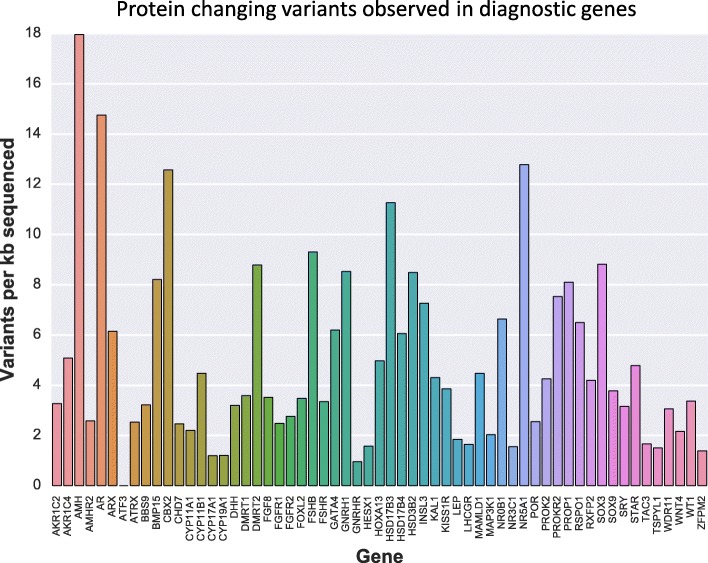
Protein changing variants seen per kilobase sequenced for diagnostic genes. A lower number of variants per kilobase sequenced suggests a higher intolerance to protein altering mutations for the gene, but may also be affected by lower ascertainment in regions that are difficult to sequence. Diagnostic DSD genes are graphed alphabetically; differing colors are used only for clarity. A small number of genes are excluded because they experienced artificially low variant counts due to technical reasons, including poor sequencing performance (*CYP21A2*, *CDKN1C*, *LHX3*), omission from sequencing in some samples (*CYB5A*), or difficulties in annotating variants accurately (*SRD5A2*)

We observed a high level of variability in the number of variants identified within each diagnostic gene. When we considered the number of protein-changing variants per kilobase for each diagnostic gene we found that some appear highly constrained and tolerate little protein changing variation, while others appear to tolerate more variation (Fig. 2).

### The targeted gene panel delivers a high genetic diagnostic rate in 46,XY DSD

Sequencing was carried out on the total cohort (455 individuals). In total we found 28,785 observations in diagnostic genes including recurrent variants; 2016 of these were protein changing and rare (<1% minor allele frequency in ESP6500, and 1000 Genomes Project), meaning that, on average, each patient had around four diagnostic gene variants. These were further filtered for frequency in our database, inheritance, and quality/depth (see “Methods”). Remaining variants were curated in accordance with previous publications using MPS analysis of DSD cohorts [8, 10] (see “Methods”), which were based on the American College of Medical Genetics and Genomics (ACMG) guidelines [15]. Rare variants in a clinically relevant DSD gene are reported here if our curation processes classified them as pathogenic, likely pathogenic, or variants of uncertain significance (VUS; not predicted to be damaging or the affected gene has not been previously reported with the described phenotype). Only variants classified as pathogenic or likely pathogenic are considered a “genetic diagnosis” in accordance with guidelines.

**Fig. 3:**
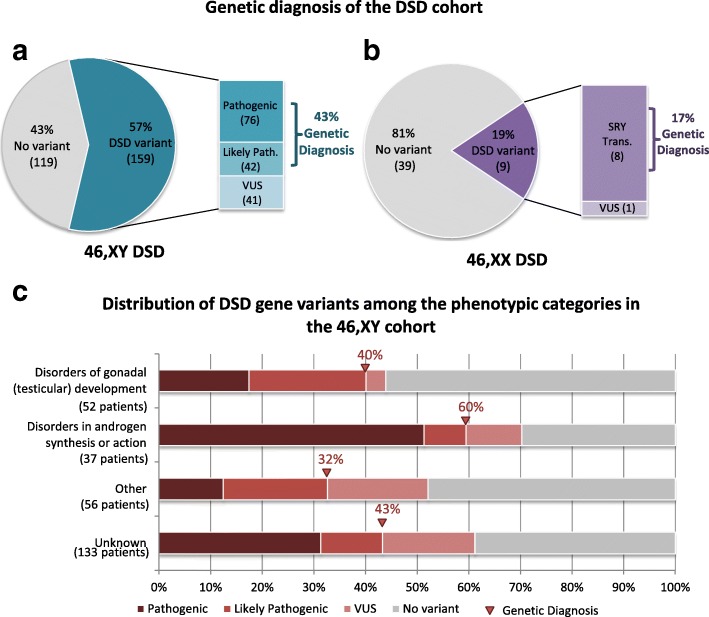
Genetic diagnosis of the DSD cohort. **a** Proportion of 46,XY DSD patients with a curated variant in a known DSD gene. In 46,XY DSD patients (278 patients), a DSD variant was identified in 57% (159 patients) of the study cohort. This was made up of 76 pathogenic variants and 42 likely pathogenic variants, resulting in a diagnostic rate of 43%. A total of 41 VUS were also found. **b** In the 46,XX DSD patient cohort (48), only 19% (9) were found to have a variant in a DSD gene, most of which were SRY translocations (8). This resulted in a diagnostic rate of 17%. **c** Distribution of curated variants in DSD genes among the 46,XY DSD phenotypic categories. Variants in a diagnostic DSD gene found to be pathogenic or likely pathogenic are considered to be a genetic diagnosis. The diagnostic outcome for each of the phenotypic categories is indicated. Disorders of gonadal (testicular) development patients had a total of 21 out of 52 patients with a pathogenic or likely pathogenic DSD variant (40%) and only two patients with a VUS (4%). Of the patients with a suspected disorder of androgen synthesis and action, 22 patients of 37 had a diagnostic variant (60%) and four had a VUS (10%). Of patients in the 46,XY other category (including hypospadias), just 18 out of 56 had a diagnostic variant (32%), with 11 patients having a VUS (19%). Finally, in the broad category 46,XY DSD unknown, which includes 133 patients, 57 had a pathogenic or likely pathogenic (43%) variant, while 24 patients had a VUS (18%). In cases where a patient had variants in multiple genes, the variant with the highest classification (pathogenic > likely pathogenic > VUS) was taken into consideration for this chart

In the 46,XY DSD cohort (278 patients) we found a total of 159 individuals (57%) had a variant in a clinically relevant DSD gene (Fig. 3a, Table 2). Of these, 76 had a pathogenic variant (48%), 42 had a likely pathogenic variant (26%), and 41 had a VUS (26%) (Fig. 3a). Thus, our panel delivered a probable genetic diagnosis in 43% of individuals affected by 46,XY DSD (the genetic diagnosis rate). The targeted gene panel proved less well suited for those affected with 46,XX DSD. Only nine of the 48 patients with 46,XX DSD had a DSD variant (Fig. 3b, Table 2), eight of which showed the presence of *SRY* material, suggesting a Y-translocation had occurred, which explained the patient’s phenotype. One patient carried a VUS. Our screen provided little insight into the basis of the DSD in the 46,XX patients, who were confirmed *SRY-*negative; they were thus excluded from the rest of the analyses. All curated variants are presented for each patient in Additional file 1: Table S1.

A large and diverse DSD cohort enabled us to determine the proportion of genetic diagnoses across the various subgroups of 46,XY DSD patients (Table 2). Of the 278 patients with a 46,XY DSD, we were able to define a genetic diagnosis in 40% of those with a disorder of gonadal (testicular) development, 60% of those with a disorder of androgen synthesis and action, 32% of those classified as “other”, and 43% of patients with an unknown 46,XY DSD (Fig. 3c, Table 2). Although our screen performs particularly well for patients with a 46,XY DSD caused by a hormonal abnormality, a large proportion (16 of 23 variants, 70%) of the identified variants had previously been reported in DSD. While the genetic diagnosis rate was lower in patients with a disorder of gonadal (testicular) development, only 33% of these variants (6 of 18 variants) had been previously described in DSD. This is the first time a large cohort of individuals affected with 46,XY DSD has been classified into distinct subsets to provide insight into the genetic etiology. This represents a dramatic improvement over current methods.

Patients in our cohort have been recruited from 12 countries. To investigate whether our panel is informative for different global regions, we grouped patients into Asia, Australia/NZ, or Europe. Each region showed a similar proportion of patients with a DSD gene variant; however, the diagnostic rate varied between regions from 33% (58 of 174 patients from Asia) to 45% for Australia/NZ (41 of 90 patients) (Additional file 2: Figure S1). This likely reflects inclusion of a larger number of patients with hypospadias from Asia, a DSD category in which the genomic basis is poorly understood (and in which environmental factors may play a role; reviewed in [16]). Nevertheless, our panel provides an improved genetic diagnostic rate in all regions.

### Variants identified in 28 diagnostic genes causative for 46,XY DSD

**Fig. 4:**
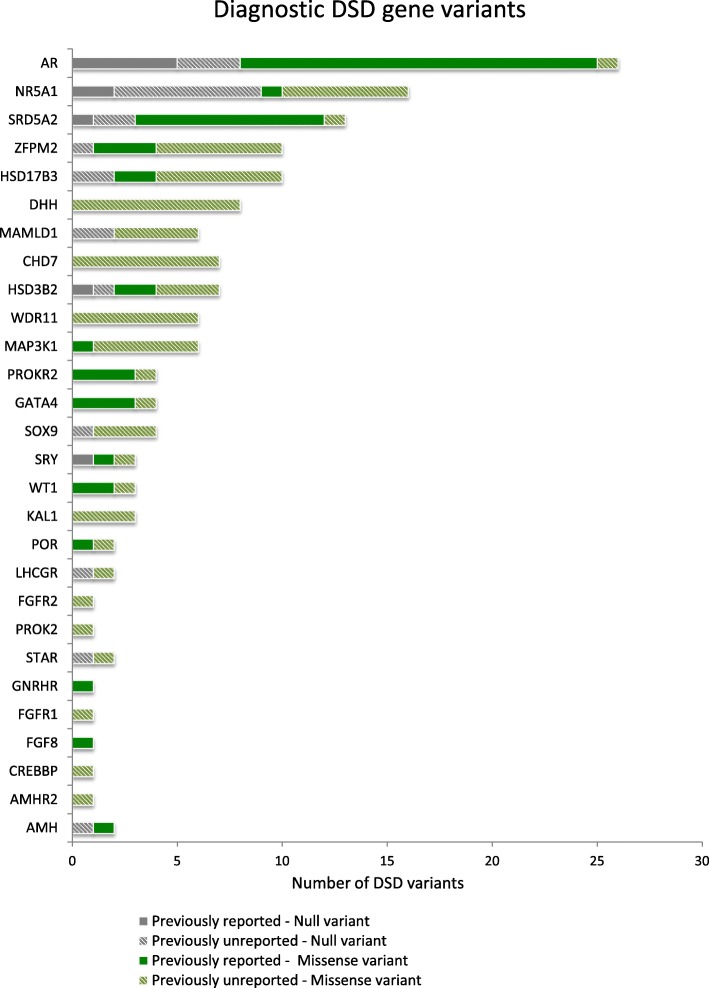
Reportable DSD variants identified in patients with 46,XY DSD. Variants were identified in 28 of a total of 64 diagnostic DSD genes. The number of previously reported (as disease causing) and unreported changes found in each diagnostic DSD gene as well as the type of change identified (missense or null variants) are shown (all variants can be found in Additional file 1: Table S1). The total number of variants is shown for each gene. The clinical relevance of each variant was checked in ClinVar, HMGD, and OMIM databases and for prior publication in PubMed

In our 46,XY DSD cohort, a total of 187 rare changes were identified in clinically relevant DSD genes. Of these, 22 occurred recurrently within our cohort. Therefore, in total we identified 151 unique variants in 28 known DSD genes (Table 1, Fig. 4). More than half of these unique variants (62%) had not been previously reported in association with a disorder (in ClinVar, the Human Gene Mutation Database (HMGD), Online Mendelian Inheritance in Man (OMIM), or published in PubMed), including 23 null and 70 missense changes (Additional file 1: Table S1, Fig. 4).

Variants in the *AR* gene were the most common (Fig. 4) with 26 unique variants curated. The majority of these were classified as pathogenic (23 variants, 86%) as they were null mutations (eight variants) or had been previously reported in association with a DSD phenotype (20 variants) (Fig. 4; Additional file 1: Table S1). *AR* has several highly repetitive tracts in exon 1 (GGN and CAG tracts). Reductions or expansions of these tracts have been suggested to contribute to numerous conditions, including hypospadias [17–19] and undervirilization [20]. We often observed patients with changes in these genomic regions compared to the reference sequence, although in many cases a proper validation of the repeat number was not possible due to sequencing technology. Thus, while we have identified these variants in patients, we have labeled them as VUS-3.

*NR5A1* and *SRD5A2* had the second and third highest number of variants called (16 and 13, respectively). Despite the preponderance of *NR5A1* publications associated with DSD, the majority of the variants we found in *NR5A1* had not been previously described (81%), including seven null and six missense variants (Fig. 4). Conversely, the majority of variants identified in *SRD5A2* (77%) were previously reported and a large proportion of them occurred recurrently within our cohort (Fig. 4; Additional file 1: Table S1).

Of interest, we identified eight unique variants in *DHH*, all previously unreported. These were all classified as damaging missense mutations with unknown inheritance, three were heterozygous, two were detected as homozygous, and two patients had two variants,potentially as compound heterozygotes. A striking number of variants were identified in *ZFPM2* (11 variants in ten patients) and *MAP3K1* (six variants in 11 patients). Both of these genes have only been described in a limited number of DSD cases [21, 22]. Three *ZFPM2* variants found in our study had been previously reported as pathogenic variants in congenital heart disease [23], although they have not been reported to be associated with genital anomalies. In the case of *MAP3K1*, the majority of variants were unreported; however, three of these variants were observed in more than one patient with 46,XY DSD (Fig. 4; Additional file 1: Table S1).

### Identifying oligogenic variants

Interestingly, a total of 13 46,XY DSD patients had more than one curated variant in a diagnostic DSD gene. Eight of these patients were classified as 46,XY DSD origin unknown and five had hypospadias (Additional file 1: Table S1, see patient IDs marked with as asterisk). Of the eight patients with 46,XY DSD origin unknown, five individuals had a known variant in *AR* in combination with another DSD gene variant; in two patients this was a pathogenic variant in an additional DASA gene (*SRD5A2* and *HSD17B3*) and in the other three it was a variant in a testis development gene. Three individuals had a pathogenic variant in a testis development gene (*MAP3K1*, *ZFPM2*, and *NR5A1*) in combination with a less damaging DSD gene variant (Additional file 1: Table S1).

Of the five patients with hypospadias, three were found to have a likely pathogenic variant in a testis development gene (*MAP3K1* and *ZFPM2*) in combination with a VUS in an additional DSD gene, while one patient had two pathogenic variants, one in a DASA gene (*HSD3B2*) and the other in a congenital hypogonadotropic hypogonadism (CHH) gene (*GNRHR*). In most cases with oligogenic inheritance, at least two of the genes were predicted to be pathogenic and/or contribute to the phenotype.

### Similar diagnostic rate in patients sequenced as singletons or trios

**Fig. 5:**
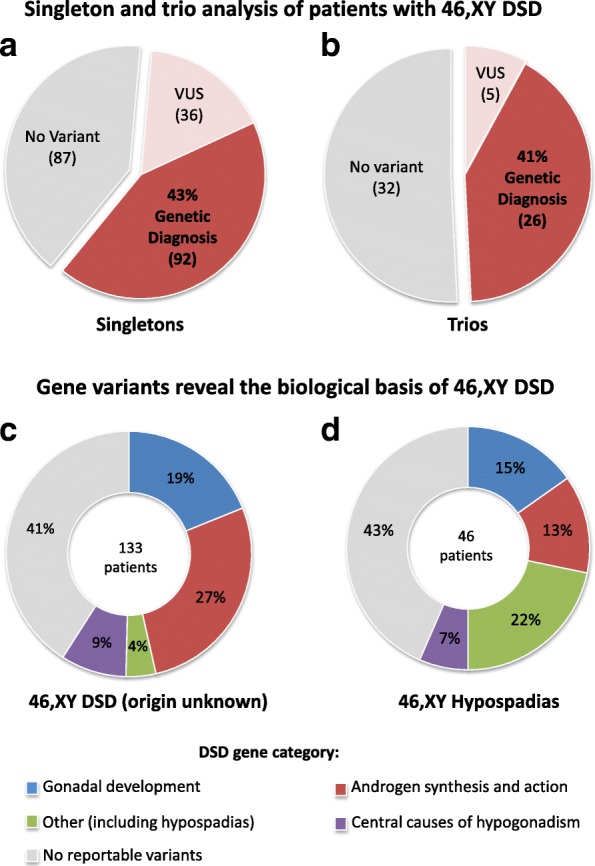
Analysis of the 46,XY DSD cohort: singletons versus trios and patients with a DSD of unknown origin. **a**, **b** Singleton or trio analysis of patients with 46,XY DSD. Individuals with 46,XY DSD were either analyzed as **a** singletons (215 patients) or **b** trios/duos. The proportion of patients with a DSD variant was higher for singletons than for trios: 68% (128 patients) versus 50% (31 patients). Singletons and trios had a similar genetic diagnostic rate (pathogenic or likely pathogenic variant) at 43 and 41%, respectively. A higher proportion of singletons had a DSD variant classified as VUS (17% of all variants in singleton) compared to trios (8% in trio analyses). **c**, **d** Gene variants reveal biological basis of 46,XY DSD. Only limited clinical information was often available for 133 origin unknown patients (**c**) and 46 hypospadias patients (**d**). Based on their curated DSD variants, these patients have been assessed on the categories of DSD gene function. In cases where a patient had variants in multiple genes, the variant with the highest classification (pathogenic > likely pathogenic > VUS) was taken into consideration. Variants annotated VUS were also included in this analysis

We have sequenced 215 patients with 46,XY DSD as singletons and 63 patients as part of a trio/duo or with a sibling. In singleton patients 128 of 215 (60%) had a variant in a diagnostic DSD gene, and for trios 31 out of 63 (43%) had a DSD variant (Fig. 5a, b). However, a likely genetic diagnosis (individuals carrying a pathogenic or likely pathogenic DSD variant) was found in 41% (26 of 63) of patients sequenced as a trio and 43% (92 of 216) of patients sequenced as a singleton (Fig. 5a). A higher proportion of singleton patients had a VUS (36 of 215, 17%) compared to trios (5 of 63, 8%). This may reflect our inability to determine variant inheritance in singletons that would have led to discounting rare familial changes. Overall, the similar genetic diagnostic rate suggests that targeted sequencing of family members alongside patients is not essential to reach an acceptable genetic diagnosis in many cases of DSD.

### Familial cases of DSD

We had seven familial cases of DSD in our cohort. Three of these had a variant in a DSD gene: patients 238 and 239 are twins with hypospadias, both of whom had a *WDR11* VUS; patients 112 and 223 (father and son, both with hypospadias) had a novel *NR5A1* frameshift mutation; patients 33 and 34 were 46,XY DSD patients with a reported pathogenic variant in *SRD5A2* (Additional file 1: Table S1). In the other four familial cases no DSD genetic variant was found with the current analysis.

### Discrepancy between phenotype/genotype and genetic clues for DSD of unknown origin

Due to the difficulty of diagnosing DSD patients, it is often challenging to apply an appropriate DSD classification to the presenting phenotype. In some cases our molecular diagnosis was at odds with the original clinical DSD classification and allowed us to suggest a reclassification, which could potentially inform clinical management. For example, patient 42 was initially described clinically as having partial androgen insensitivity, but was found to have a heterozygous *DHH* variant. As our molecular diagnosis differed from the original clinical classification, we classified this variant as VUS-2 (predicted pathogenic but does not fit phenotype; Additional file 1: Table S1); therefore, further investigation is warranted.

In cases with limited phenotypic descriptors, genetic analysis pointed to a more concise DSD classification. This was performed on two groups of individuals, those with 46,XY DSD unknown origin (undervirilization category) and those with “isolated hypospadias”. The first group (133 patients) consisted of the following: limited clinical information, noted to have ambiguous genitalia, undervirilization phenotypes including hypospadias, bifid scrotum, micropenis, cryptorchidism, often with no further description of either internal structures or hormonal levels. When we reassessed this group by the type of DSD variant identified, a significant proportion had variants in genes known to cause disorders of androgen synthesis and action (36 patients, 27%) or disorders of gonadal (testis) development (25 patients, 19%) (Fig. 5c), highlighting the potential genetic basis of their phenotype.

Our cohort also included 46 patients with 46,XY DSD who were defined as having isolated hypospadias. Again, this group of individuals were often referred with limited clinical information. While ten of these patients (22%) did have a variant in a gene known to cause isolated hypospadias, six of the 46 patients (13%) had a variant in an androgen synthesis or action gene and seven (15%) had a variant in a gonadal (testis) development gene (Fig. 5c).

### Relevance of CHH variants in 46,XY DSD

One interesting observation limited to both 46,XY origin unknown and isolated hypospadias groups was that 9% of patients carried a variant in a known CHH/Kallmann syndrome gene (a total of 16 patients; Fig. 5b, c. In general, variants in CHH genes were rarely detected in patients outside these groups (two other patients in total). Variants were found in seven CHH genes (*CHD7*, *KAL1*, *WDR11*, *PROK2*, *PROKR2*, *FGF8*, and *FGFR1*; Additional file 1: Table S1). Five variants have been previously reported as pathogenic in CHH, with a number of these showing decreased activity in functional studies (e.g., FGF8 p.P26L, PROKR2 p.S188L and p.L173R) [24–26]. Of the previously unreported variants, 18 were predicted to be pathogenic by the in silico models used but were classified as VUS-2 as the spectrum of phenotypes seen in these patients does not correlate with a usually less severe CHH phenotype. It is interesting to speculate that these variants in the CHH genes may be contributing to 46,XY DSD phenotypes.

## Discussion

DSD are a major pediatric concern, estimated to occur in 1.7% of all live births [27]. Providing a molecular diagnosis for these patients is often difficult given the large heterogeneity of clinical presentations included in this group of disorders. A previous study has stated that a clinical genetic diagnosis is only made in 13% of all DSD patients in a hospital setting [7]. In particular, 46,XY DSD are not well diagnosed at the molecular level. However, MPS is now fast becoming a standard assay for molecular diagnosis of rare Mendelian disorders and has been successfully used on small cohorts of DSD patients [7, 8, 10]; in particular, a research study of 40 cases using WES provided a likely genetic diagnosis in 35% [8]. We present a MPS targeted DSD gene panel on one of the largest collections of 46,XY DSD reported to date (278 patients). Our data provide an improved genetic diagnostic rate of 43% for these individuals. Targeted panel sequencing offers many advantages over WES or WGS. It is an economically viable option as reagent costs (AUD$300 for our panel) and curation times are reduced and the chances of incidental findings are negligible. Given that WES sequencing is not currently funded by government or private health care providers in Australia and other jurisdictions, we propose our targeted DSD gene panel should be considered as a first tier test in the clinical diagnosis and management of 46,XY DSD patients.

### MPS evaluation

The capacity of a targeted gene panel as a diagnostic tool is underpinned by its performance in diagnostic gene sequencing. For the 64 diagnostic DSD genes we observe almost complete coverage by our targeted gene panel, with 99.4% of bases covered by at least one amplicon, and 97.2% of bases covered by at least two amplicons. Despite the coverage by amplicons, we observed significant regions over some diagnostic genes that were covered by reads at less than acceptable levels for diagnostic use. In the case of *CYP21A2* this was attributable to the presence of a pseudogene having high homology with the target gene. Such genes are extremely difficult to interrogate with any technology in which short reads are used due to the inability to uniquely map reads to these locations. As such, the failure is not specific to the HaloPlex technology we used for our targeted gene panel, but relates to current MPS technology in general. Other shortcomings were attributable to the distinctive characteristics of the HaloPlex assay. For example, the propensity for individual amplicons to sporadically fail to produce reads requires that care be taken during the targeted capture design to ensure important regions are covered by multiple amplicons.

Overall, the effective targeting efficiency of our targeted gene panel was comparable to that of other systems for targeted enrichment, with between 60 and 70% of base reads generated from the targeted regions. Despite some of the drawbacks associated with all current MPS technologies, our analysis has shown that a targeted panel can form a powerful diagnostic tool.

### A large international cohort of patients with DSD

For this study, we compiled DNA from 326 patients and 129 family member participants, making this the largest reported cohort of patients with DSD. We have shown that our MPS targeted DSD gene panel is useful for the identification of diagnostic variants in a wide range of 46,XY DSD, and a likely genetic diagnosis was achieved in 43% of cases. It is interesting to note that prior to their inclusion in our study, a large proportion (at least 30% to our knowledge) of the patients had undergone genetic pre-screening (such as single-gene Sanger sequencing or microarrays), which ultimately affects our overall diagnostic rate. This suggests that if applied as a first-tier diagnostic test, we could expect our panel to provide an even greater diagnostic outcome. Our results support previous conclusions reached by others [7, 8, 10] indicating that diagnosis of 46,XY DSD can be significantly enhanced through use of MPS technologies, albeit on a much larger scale.

Our highest diagnostic rate of 60% (22 of 37 individuals) is for patients who have disorders of androgen synthesis and action. A large proportion of these patients had variants previously described in DSD (17 of 22, 77%), primarily variants in *AR* and *SRD5A2*. The publically available *AR* database has a total of 546 unique entries (this includes recurrent variants associated with different phenotypes), with 339 of them associated with DSD [28]. Of the 26 unique *AR* variants found in our 46,XY DSD cohort, only six were previously unreported (four null mutations and two missense), suggesting the vast majority of DSD-causing *AR* variants have been defined.

Large-scale MPS sequencing has not been previously reported for 46,XX DSD; therefore, we analyzed 48 patients with various forms of 46,XX DSD to determine how a targeted gene panel would perform. We found that gene panel testing is not informative for 46,XX DSD in its current format. The majority of 46,XX DSD patients included in our study were reported to have had a prior test to examine gain of *SRY*. We independently identified eight patients carrying *SRY* (indicative of translocation) from our 46,XX DSD cohort. Translocation of *SRY* accounts for approximately 80% of individuals with 46,XX testicular DSD [29]. The majority of other reports describing the molecular basis for disorders of ovarian development are copy number variants (CNVs) in a number of testis-promoting or ovary-promoting genes (for example, *SOX9* [30–33], *FGF9* [34], RSPO1 [35, 36], WNT4 [37, 38]; reviewed in [20]). A recent study showed the contribution of small exon level deletions in Mendelian disease has been underestimated [21], highlighting the need for similar analyses in 46,XX DSD. Further work to assess the ability of our targeted gene panel in detecting CNVs is underway.

### Identification of variants: prevalence in disorders of gonadal (testicular) development

This study has allowed us to identify a total of 76 pathogenic, 42 likely pathogenic variants, and 41 VUS in known DSD genes, more than half of which were previously unreported. This substantially expands our current knowledge of diagnostic DSD variants. In a study on DSD patients using WES, Baxter et al. [8] identified a number of patients with variants in *MAP3K1*, a gene previously associated with 46,XY CGD [22]. Similarly, we found 11 patients with heterozygous variants in *MAP3K1* representing six separate variants. Interestingly, a variant we detected in two patients with 46,XY CGD (p.L189R) had been previously reported in individuals with a similar phenotype [22].

We also observed two *MAP3K1* variants (p.M312L and p.A1443V) that recurred in multiple patients who presented with a diverse range of phenotypes (including CGD, PGD, hypospadias, and undervirilization). This suggests variants in this gene may be associated with a greater phenotypic variability than had been previously thought, although population specific polymorphisms may be involved with the less severe phenotypes. While a high level of variability between the number of variants in each diagnostic gene was observed, *MAP3K1* showed intolerance to protein changing variation compared to other genes, both in our data and also on ExAC (with a missense Z-score of 1.53 and a probability of LOF intolerance of 1). Given this, and previous reports using exome sequencing in a smaller cohort [8], we can confidently infer 10% prevalence of *MAP3K1* variants amongst 46,XY disorders of gonadal (testicular) development classification (5 of 52 patients); however, this could be up to 18% if the *MAP3K1* phenotypic spectrum is expanded. Further functional analysis will be required to fully test these previously unreported variants.

A number of studies have identified *DHH* variants in individuals presenting with a range of gonadal dysgenesis (46,XY partial GD to complete GD), with or without polyneuropathy [39–42]. The majority of these variants were homozygous, with only one report of a heterozygous single base pair deletion causing 46,XY PGD [40]. We identified seven patients with eight previously undescribed *DHH* missense variants (none were reported to have polyneuropathy). Homozygous or potentially compound heterozygous *DHH* variants were identified in four patients presenting with 46,XY DSD female phenotype, while the three individuals with heterozygous *DHH* variants had diverse phenotypes, including DASA, DSD origin unknown, and hypospadias. The clinical significance of heterozygous *DHH* variants is still unclear; however, variants in this gene can present as apparent DASA due to an impairment of Sertoli cell–Leydig cell interaction during gonad development [39]. Identifying a genetic diagnosis in *DHH* can impact clinical management due to the increased risk of gonadal malignancy in such patients [39, 40].

In humans, mutations in *ZFPM2* have commonly been shown to be associated with congenital heart disease [23] but only recently have heterozygous and homozygous missense variants been detected in individuals with isolated 46,XY PGD and CGD [21]. We identified nine *ZFPM2* missense and one frameshift mutation in six patients with 46,XY disorders of testicular development (52 patients), providing a genetic outcome for 12% of these patients.

We also observed *ZFPM2* variants in three individuals with hypospadias and in some instances this was in conjunction with another DSD gene variant that had not previously been reported. In the case of *MAP3K1*, *DHH*, and *ZFPM2* it is difficult to distinguish whether variants identified in patients categorized as isolated hypospadias expand the known mutation spectrum of these genes or whether these patients have underlying gonadal dysgenesis.

### A role for oligogenetic inheritance in DSD

A recent report suggested that the expanded DSD phenotypic spectrum associated with *NR5A1* mutations was attributed to oligogenic inheritance in other testis development genes such as *MAP3K1* [43]. Similarly we found evidence of this accumulative effect within our cohort of patients with severe hypospadias. In three of these patients we found oligogenetic inheritance of a variant in a testis development gene (*MAP3K1* and *ZFPM2*) in combination with a VUS (often in a CHH gene). Another patient (251*), also with severe hypospadias, was found to have two pathogenic variants, one in *HSD3B2* (a gene implicated in proximal hypospadias) [44] and the other in a known CHH gene, *GNRHR.* Finally, in patients with 46,XY DSD of unknown origin we found five with an AR mutation in combination with an additional variant in either androgen action or gonadal development. This suggests that like *NR5A1*, *AR* may show oligogenic involvement in DSD.

CHH leads to a reduction in gonadotrophin release from the pituitary and can present as an inability to enter puberty or even as mild undervirilization at birth in 46,XY males [45]. This has been reported to be associated with phenotypes such as cryptorchidism and micropenis, but is typically thought not to cause isolated hypospadias or more severe phenotypes such as ambiguous genitalia. We found a significant proportion of the patients with 46,XY undervirilization or hypospadias had predicted pathogenic or previously reported variants in genes that are known to cause CHH. This has also been seen in WES sequencing of DSD patients [8], raising the intriguing possibility that mutations in these genes may contribute to a broader base of DSD phenotypes than previously thought.

### Sequencing singletons and trios delivers a similar diagnostic rate

Where MPS is concerned, trios are often encouraged as the gold standard, to allow better variant filtering and curation. Although the total number of individuals sequenced in our study as singletons versus trios/duos was substantially different (215 versus 63), we found that the proportion of patients with a probable genetic diagnosis was similar between these groups. We observed a higher number of variants curated and deemed VUS in the singletons, variants which may not have stood up to scrutiny if the mode of inheritance was known (where familial variants are removed). Screening patients with DSD as singletons provides a cost-effective clinical genetic diagnosis that is comparable to trio analyses, although trio analysis can reduce overall curation time. Nevertheless, in a gene discovery setting, trio analysis will still be highly valuable as it eliminates rare familial variants, confirms modes of inheritance, and detects de novo events.

### Genetic screening provides clues for biological basis of DSD and clinical management

We found our panel to be highly informative for patients affected by DSD with an unknown biological basis. Given that this kind of sequencing is relatively inexpensive and quick and has a high genetic diagnostic rate, it has potential as a first-tier clinical test to help inform clinical management. A molecular diagnosis can provide clues as to the biological basis of DSD and may direct clinicians towards a specific clinical test. This could be particularly useful in situations or countries in which clinical tests such as histopathological examination, hormonal profiling, and advanced imaging are costly or not routinely performed. We have shown that our gene panel will assist with DSD classification in a situation where an in-depth clinical presentation is not available. The caveat to this is that the mutation spectrum of a number of genes encompasses multiple clinical presentations. For instance, the spectrum of *NR5A1* mutations presented in our 46,XY cohort as CGD (two patients), PGD (four patients), hypospadias (one patient) and DASA (one patient); additionally, it has also been shown to include spermatogenic failure [46]. This needs to be taken into consideration, as a patient with a variant in *NR5A1* cannot be strictly classified as having a disorder of testis development. However, genetic etiology is crucial for informing clinical management and provides insights into the diverse heterogeneous nature of DSD.

In clinical genomics, systematic classification guidelines are constantly evolving as evidence-based tools, resources, and databases become available. We followed the same process employed by previous genomic studies of DSD patients [8, 10], based on the ACMG guidelines for curation of clinical variants. Nevertheless, several limitations of our study hindered curation—the lack of parental/familial samples for many patients and, in some cases, limited clinical phenotyping. In addition, as we did not sequence unaffected control samples from each ethnic group we assayed, we relied heavily on online databases like ExAC for population allele frequencies. These may not always accurately reflect small ethnic minorities. Future adoption of our panel as an accredited clinical diagnostic test will resolve these issues for prospective cases as a more stringent variant classification would be used.

Although a success on many levels, our genetic panel did not provide answers for 39 patients with 46,XX DSD and 52 patients with 46,XY DSD where no diagnostic variant was detected. Like many sequencing technologies, there are regions in our panel that have low coverage. As we do not use alternative methods to fill these gaps, it is possible that we might miss diagnostic variants that fall within these regions. One limitation of targeted gene sequencing is that detection of CNVs is significantly more challenging than single nucleotide variants or INDELs. While a range of CNV detection methods have been developed to work with targeted sequencing data, specialized bioinformatic expertise is required to obtain accurate results. Furthermore, standard methods are generally not optimized well for use with the HaloPlex technology. CNVs are known to contribute to DSD, and our current inability to detect these in our targeted gene panel means that we may be missing diagnostic changes in DSD patients. We are currently working to create a bioinformatic pipeline designed to use these data to assay for CNVs, which will be a useful additional tool in the future.

While this study has focused on diagnostic DSD genes, our targeted panel also includes 967 candidate genes identified from animal model studies, implicated genetic pathways, and gonad RNA-seq experiments. Currently, our research group is pursuing several novel candidate genes identified from these data, although these studies are ongoing and beyond the scope of this article. Further analysis of these genes (as well as WES or WGS sequencing and microarrays on select patients) promises to reveal novel candidate genes that may contribute to the development (and disease) of the reproductive system in humans. Future detailed analysis of these genes and their function will further improve genetic diagnosis and clinical management of DSD.

## Conclusions

Our targeted DSD gene panel is an effective means of providing a genetic diagnosis for patients with 46,XY DSD (43% of cases). Employing this in a large, diverse cohort of patients with DSD has provided us with a better understanding of the underlying genetic etiology of this condition. In particular, we have expanded the range of phenotypes associated with several DSD genes. Given the rapid turn-around time and reduced cost compared to WES or WGS, we believe that this targeted gene panel could be used as a first tier clinical diagnostic test for 46,XY DSD to assist in optimizing clinical management for these patients.

## Methods

### Ethics statement

This project (Molecular genetics of sex determination and gonad development, HREC 22073) has been approved by the Royal Children’s Hospital (Melbourne, Australia) ethics committee. Patients and family members were enrolled after signing informed consent (and in the case of minors, parental consent was also obtained). For patients recruited in countries other than Australia, consent was also obtained using local ethics and consent and DNA transferred through a memorandum of understanding between the Murdoch Childrens Research Institute (MCRI) and the corresponding institute/hospital. This study was conducted in compliance with the Helsinki Declaration.

### Patient clinical data

Clinical notes were collected for each patient during their standard clinical care by trained clinicians, and these data were transferred to us under the informed consent (HREC 22073). This often included a description of their external genitalia, internal reproductive organs, hormonal profile, and additional notes of interest (i.e., additional anomalies or family history). All of the patients had undergone karyotyping. Many patients had previous clinical microarrays and or *SRY* screening. Some had had single-gene sequencing (i.e., *AR*). Only patients that were negative for these tests were included in the cohort discussed here. De-identified DNA from each patient was stored in a secure DNA storage facility.

### DNA extraction

Genomic DNA extraction from EDTA-blood samples was performed in an independent laboratory such as Victorian Clinical Genetics Service (VCGS), or at local hospitals. DNA quality was assessed using an Agilent gDNA ScreenTape run on 2200 TapeStation (Agilent Technologies Inc.) and concentration was measured in our laboratory on a Qubit 3.0 Flurometer using the broad range DNA quantification kit (ThermoFisher scientific).

### Targeted panel design

The targeted panel uses the Agilent HaloPlex method for sample preparation and was designed using the Agilent SureDesign software (https://earray.chem.agilent.com/suredesign/). The gene panel currently includes a total of 1031 genes, microRNAs, and potential regulatory regions. These targeted regions comprise 64 known diagnostic genes for DSD (Table 1), potential DSD candidate genes from human and animal studies, as well as whole pathways with one or more genes being associated with DSD (capture size 2.5 Mb).

### Targeted gene panel library preparation

Library preparation was carried out according to the manufacturer’s instructions, with the exception that half reactions were performed. Briefly, genomic DNA (125 ng gDNA) was digested with 16 different restriction enzymes at 37 °C for 30 min to create a library of gDNA restriction fragments. Both ends of the targeted fragments were then selectively hybridized to biotinylated probes from the HaloPlex DSD panel (Agilent Technologies Inc.), which resulted in direct fragment circularization. During the 16-h hybridization process, HaloPlex Illumina Barcodes were incorporated into the targeted fragments. Circularized target DNA–HaloPlex probe hybrids containing biotin were subsequently captured by HaloPlex magnetic beads on the Agencourt SPRIPlate Super magnet magnetic plate. DNA ligase was added to close the nicks in the hybrids and freshly prepared NaOH was used to elute the captured target libraries.

The target libraries were then amplified and purified using AMPure XP beads. Amplicons ranging from 150 to 550 bp were finally quantified using an Agilent D1000 DNA ScreenTape on the 2200 TapeStation to validate the enrichment of the libraries.

MPS was carried out according to the manufacturer’s instructions at the Translational Genomics Unit at the Murdoch Childrens Research Institute /VCGS, using either the Illumina MiSeq, NextSeq500, or HiSeq4000 or at the Centre for Translational Pathology, The University of Melbourne using an Illumina HiSeq2500. For the case of MiSeq samples, paired-end 2 × 150-bp reads were used, while the HiSeq 2500 produced 2 × 150-bp reads.

### Bioinformatic analysis

The sequencing data were analyzed using Cpipe, an exome analysis pipeline designed at MCRI [47]. Cpipe was customized to improve performance on HaloPlex data in several ways. The reads were first trimmed using an in-house trimming method specialized for HaloPlex reads. The custom trimming method detects contamination by matching the expected sequence that will be observed when adapter sequence appears adjacent to the known amplicon boundaries in the sequencing data. The trimmed reads were aligned using the BWA mem [48] alignment algorithm, followed by base quality score recalibration and local realignment around INDELs using the Genome Analysis Toolkit (GATK) [49]. Notably, deduplication of reads was not applied, consistent with Agilent recommendations for processing HaloPlex data. This requirement stems from the properties of HaloPlex data in which reads appear in tall towers sharing identical start and end positions. Such reads are falsely considered to be PCR duplicates by deduplication software, and thus deduplication causes a severe and unnecessary loss of read coverage depth. Variants were called using the GATK UnifiedGenotyper and annotated using a combination of SnpEFF [50] and Annovar [51] to predict protein changes, population frequencies and add other functionally informative data about each variant. The customizations to Cpipe (Cpipe version 2.1) and specialized trimming software are available at https://github.com/ssadedin/halo_dsd and in Zenodo (64133851; https://zenodo.org/badge/latestdoi/64133851).

Four in silico models, SIFT [52], Polyphen2 [53], LRT [54], and Mutation Taster [55], were used as well as GERP++ in some cases [56]. Manual mapping of the genomic changes and submission to the in silico tool was performed for variants identified in *SRD5A2* as the transcript was retired (transcript reference NM_000348.3).

#### Variant filtering and curation

##### Frequency

Variant files were filtered to include only rare (1000 Genomes Project ≤0.01 and ESP5,400 or ESP6,500 ≤0.01), functional variants (different ESP databases reflect updates during our analysis). As we did not run control samples from each ethnic subgroup, the allele frequency of population subgroups most reflecting the ethnic background were checked on ExAC and variants discounted if they were common (>0.01). In addition to public databases, the variant frequency within our cohort (as a total database call and a frequency per sequencing run) was also tracked. This allows us to identify variants that may be the result of amplification or sequencing error (which are common in one sequencing run), or that may be common in a subpopulation but not well represented on publically available databases (i.e., Indonesian). Thus, we also discounted variants found in greater than 15 samples in the same run or in the total database. Following this, only variants in known diagnostic DSD genes (64) were considered.

##### Variant quality/depth

Variants in known DSD genes were evaluated for coverage depth and read quality and also visually inspected using the Integrative Genomics Viewer (http://www.broadinstitute.org/igv/). In some cases of low coverage or depth, validation by Sanger sequencing using the standard protocol for BigDye® Terminator v3.1 Cycle Sequencing Kit (Life Technologies, Carlsbad, CA, USA) was carried out at the Centre for Translational Pathology, The University of Melbourne.

##### Inheritance

If the inheritance mode did not fit with the described phenotypic/genotypic spectrum, then the variant was not considered for further curation. For trios and families, different additional filters were applied to distinguish between de novo, maternally or paternally inherited, and compound heterozygous genetic models.

##### Variant curation

Variants previously reported to cause disease in OMIM, ClinVar, HMGD, and PubMed searches were hereafter called as “reported”. Each variant was then classified according to the following curation guidelines. Pathogenic variants are null mutations, such as frameshifts, deletions, premature stop codons, and splice site mutations, in genes where a loss of function is a known disease mechanism and where the described phenotype correlated with the patient’s. Alternative transcripts and splice site variations were taken into account. Missense variants previously found in a patient with a similar clinical presentation were also considered pathogenic variants. Likely pathogenic variants are novel missense variants in known DSD genes that fit the phenotype, had the correct inheritance pattern, and are predicted to be damaging in greater than three of our four in silico prediction tools. The remaining variants were of unknown significance (VUS). These were further separated into VUS-1 (within the disease spectrum/fit clinical notes but predicted benign), VUS-2 (predicted deleterious (in at least three of four in silico predictors) yet not within the known spectrum of phenotypes), or VUS-3 (if they fell within the region of CAG or GGN repeats in the AR receptor, regions of which the relevance in DSD is as yet unclear).

## Additional files

Additional file 1: Table S1. DSD gene variants. Each variant found in a diagnostic gene (after the filtering and curation process) is shown. In some cases where the gene is inherited in an autosomal recessive manner, two variants are grouped together. Inheritance has been indicated where familial samples were available: *negative* indicates negative for variant and *N/A* sample not available. De novo events have only been noted where both parental samples were available and found to be negative for the change. *Previously reported* refers to a variant being described in either ClinVar, HGMD, or a publication in a peer-reviewed journal via a PubMed search. Variants were classified consistent with previous MPS publications of DSD cohorts [8, 10] which were based on ACMG guidelines [15]. VUS were called for three reasons: 1 = fits phenotype but predicted to be benign; 2 = damaging but doesn’t fit phenotype; or 3 = variant in the *AR* repetitive region. Patients marked with an asterisk were identified to have two or more diagnostic gene variants. Null variants (frameshifts, splice sites mutations, and premature stop codons) are shown in *bold*. Patients have been classified based on clinical notes provided, according to the recommended classification of DSD in the Chicago consensus report. Classifications: *CGD* complete gonadal dysgenesis, *DASA* disorders of androgen synthesis or action, *DSD* DSD of “unknown” origin; hypospadias, *LCH* Leydig cell hypoplasia, *OT* ovotesticular DSD, *PGD* partial gonadal dysgenesis, *PMDS* persistent Müllerian duct syndrome; syndromic, *T* testicular DSD. Related affected individuals are indicated. File is in Excel spreadsheet format. (XLSX 47 kb)
Additional file 2: Figure S1. DSD gene variants in different global regions. DSD gene variants among the international cohort of 46,XY DSD patients. For ease of analysis, countries were grouped together into regions: Asia comprises Indonesia (97), Pakistan (25), Vietnam (35), Cambodia (16), India (1), a total of 174 patients ; Europe comprises the Netherlands (38), Austria (15), Belgium (6), and Italy (2), a total of 61 patients; and AUS & NZL comprises Australia (83) and New Zealand (7), a total of 90 patients. All curated variants are shown; those which have been curated and called pathogenic, likely pathogenic, and VUS. In the cohort from Asia, 35% of the patients were found to have a diagnostic variant (pathogenic or likely pathogenic), while this was 44% for Europe and 45% for AUS/NZL. Two patients from Canada were not included in the diagram. (PPTX 158 kb)

## Abbreviations

ACMG: American College of Medical Genetics and Genomics
AR: Androgen receptor
bp: base pair
CAH: Congenital adrenal hyperplasia
CGD: Complete gonadal dysgenesis
CHH: Congenital hypogonadatropic hypogonadism
CNV: Copy number variation
DASA: Disorders of androgen synthesis or action
DSD: Disorder of sex development
gDNA: genomic DNA
HMGD: Human Gene Mutation Database
MPS: Massively parallel sequencing
MRKH: Mayer-Rokitansky-Küster-Hauser syndrome
OMIM: Online Mendelian Inheritance in Man
OT: Ovotesticular
PCR: Polymerase chain reaction
PGD: Partial gonadal dysgenesis
T: Testicular
VCGS: Victorian Clinical Genetics Service
VUS: Variant of unknown significance
WES: Whole exome sequencing
WGS: Whole genome sequencing.
