# Supplementary material for: Disorders of sex development: insights from targeted gene sequencing of a large international patient cohort
Source: Genome Biol. 2016 Nov 29;17:243. doi: 10.1186/s13059-016-1105-y (PMC5126855; doi:10.1186/s13059-016-1105-y)
Supplement: Additional file 2: Figure S1. — DSD gene variants in different global regions. DSD gene variants among the international cohort of 46,XY DSD patients. For ease of analysis, countries were grouped together into regions: Asia comprises Indonesia (97), Pakistan (25), Vietnam (35), Cambodia (16), India (1), a total of 174 patients ; Europe comprises the Netherlands (38), Austria (15), Belgium (6), and Italy (2), a total of 61 patients; and AUS & NZL comprises Australia (83) and New Zealand (7), a total of 90 patients. All curated variants are shown; those which have been curated and called pathogenic, likely pathogenic, and VUS. In the cohort from Asia, 35% of the patients were found to have a diagnostic variant (pathogenic or likely pathogenic), while this was 44% for Europe and 45% for AUS/NZL. Two patients from Canada were not included in the diagram. (PPTX 158 kb) [file 13059_2016_1105_MOESM2_ESM.pptx]

## Slide 1
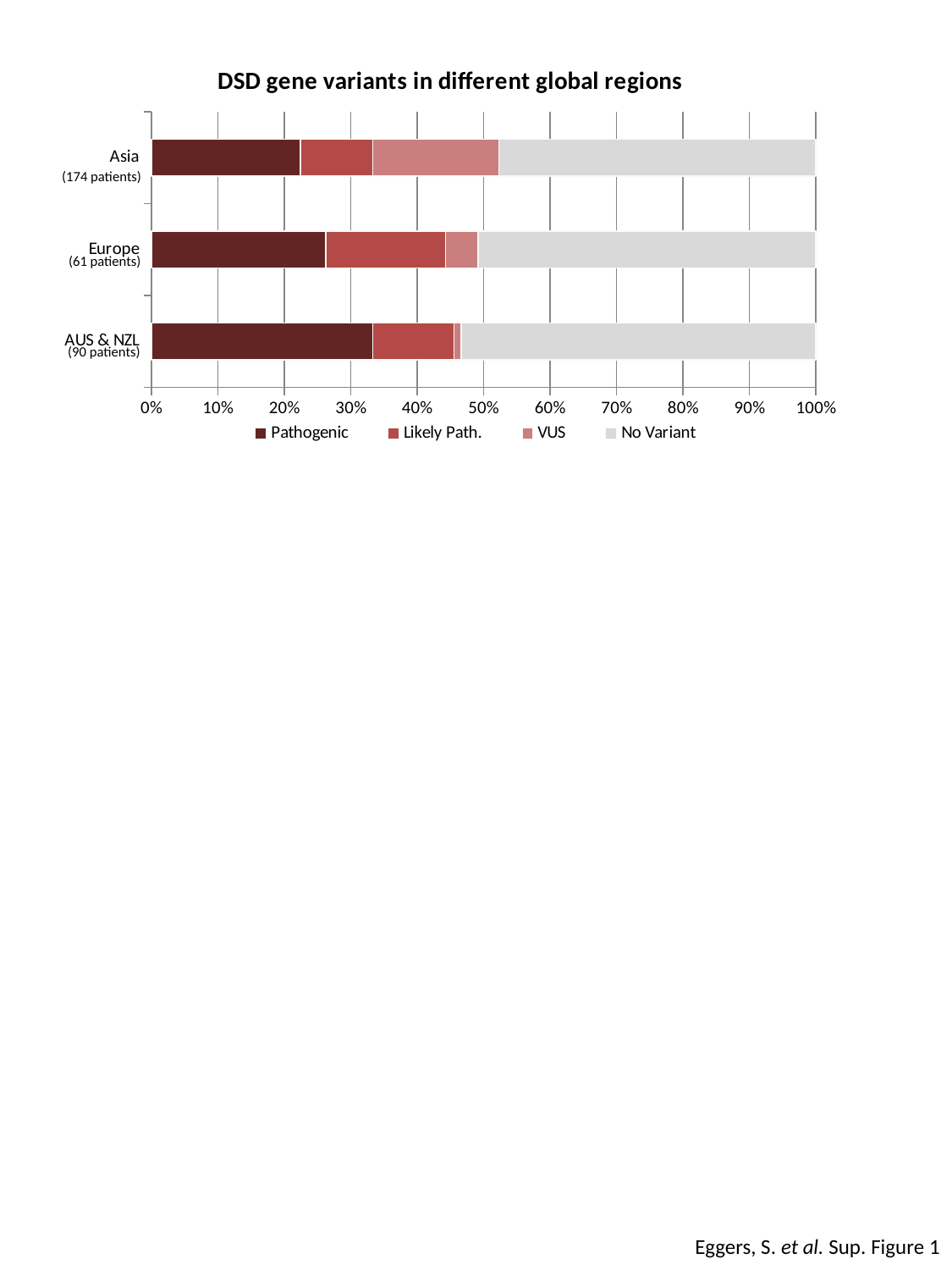

### Chart: DSD gene variants in different global regions
| Category | Pathogenic | Likely Path. | VUS | No Variant |
|---|---|---|---|---|
| AUS & NZL | 30.0 | 11.0 | 1.0 | 48.0 |
| Europe | 16.0 | 11.0 | 3.0 | 31.0 |
| Asia | 39.0 | 19.0 | 33.0 | 83.0 |(174 patients)
(61 patients)
(90 patients)
Eggers, S. et al. Sup. Figure 1
